# A survey of cranberry fruit rots in commercial production beds in Oregon and Washington

**DOI:** 10.3389/fpls.2024.1457320

**Published:** 2024-09-19

**Authors:** Don C. Valentine, Cassie Bouska, Virginia O. Stockwell

**Affiliations:** ^1^ Department of Botany and Plant Pathology, Oregon State University, Corvallis, OR, United States; ^2^ Department of Horticulture, Oregon State University Extension Service, Myrtle Point, OR, United States; ^3^ United States Department of Agriculture, Agricultural Research Service, Horticultural Crops Disease and Pest Management Research Unit, Corvallis, OR, United States

**Keywords:** *Vaccinium macrocarpon*, Stevens cranberry, fungal pathogens, *Coleophoma*, *Colletotrichum*, *Physalospora*, *Neofabraea actinidiae*

## Abstract

**Introduction:**

Fungal fruit rots are a perennial threat to the production of cranberries. Eleven genera of fungi have been reported to cause cranberry rot in the field and/or during cold storage. Oregon and Washington rank fourth and fifth in the production of cranberries in the USA, but much of the research on cranberry fruit rots has been conducted in Wisconsin, New Jersey, and Massachusetts.

**Objective:**

The primary objective of this project was to describe the current composition of cranberry fruit rot complex in Oregon and Washington.

**Methods:**

A survey of fungal fruit rot pathogens was conducted over four years in commercial cranberry farms located in the Pacific Ocean coastal zone in Oregon and Washington.

**Results:**

Yield, rot incidence, and fungal pathogens isolated varied year-to-year. Pathogens isolated frequently from field-rotted cranberries included the cranberry fruit rot genera described in other cranberry production regions of the USA, such as Colletotrichum, Coleophoma, and Physalospora. Neofabraea actinidiae, a recently described cranberry fruit rot, was isolated consistently from field-rotted cranberries from beds with specific fungicide usage patterns. N. actinidiae also was one of the more common storage rot pathogens in this region, alongside other well-established storage rots like Coleophoma and black rots caused by Allantophomopsis cytisporea, A. lycopodina, and Strasseria geniculata.

**Conclusions:**

These findings may have important implications for Washington cranberry production because a large proportion of the crop is dry-harvested, placed in cold storage, and then sold as fresh cranberries. Climatic differences among the cranberry production areas across the United States may affect the disease incidence and prevalence of different genera of cranberry fruit rot pathogens, as summer months in Oregon and Washington are often much cooler and dryer than in Wisconsin and east coast states and may account for differing presence of various cranberry fruit rot fungi.

## 1 Introduction

In the United States, commercial cranberry (*Vaccinium macrocarpon*, L.) production is centered in Wisconsin, Massachusetts, New Jersey, Oregon, and Washington (United States Department of Agriculture, National Agricultural Statistics Service, 2019). Oregon and Washington rank fourth and fifth, respectively, in cranberry production in the USA. Nearly all cranberry farms in Oregon use a “flood harvest” method, which entails a gentle agitation of the low-growing vines to loosen the berries followed by flooding the bed and collecting the floating berries. The cranberries are delivered to a processing plant where they are washed, sorted, and placed in cold storage until they are processed into juice, craisins, sauce, and other products. In Washington, about a third of cranberry production is dry-harvested using a mechanical picking machine (Strik et al., 2002). Dry-harvested cranberries are sold as fresh fruit, allowing for premium crop prices in comparison to processed berries (United States Department of Agriculture, National Agricultural Statistics Service, 2024). Fresh berries are typically placed in cold storage until November and December and sold during the holidays. The dry-harvested acreage makes Washington a unique cranberry-growing region, because less than 5% of cranberry production in the U.S. is destined for the fresh market and not processed after harvest (United States Department of Agriculture, National Agricultural Statistics Service, 2024).

A major concern for growers are cranberry fruit rots (“CFRs”), which are caused by at least 14 different fungal species including: *Allantophomopsis cytisporea, A. lycopodina, Botrytis* sp.*, Coleophoma cylindrospora, Colletotrichum acutatum* complex and *Colletotrichum gloeosporioides* complex sp.*, Diaporthe vaccinii* and other *Diaporthe* sp., *Godronia cassandrae, Monilinia oxycocci, Neofabraea actinidiae, Phyllosticta elongata, Phyllostica vaccinii, Physalospora vaccinii*, and *Strasseria geniculata* (Polashock et al., 2017; Valentine et al., 2024). These fungi may cause cranberry rot diseases in the field, during post-harvest storage or both. Failure to manage CFRs can lead to total crop loss (Oudemans et al., 1998).

The combination of warm humid summer weather, the low growth habit of the vines and often-damp nature of cranberry beds may provide a conducive habitat for fruit rot pathogens. With optimal cultural practices and use of fungicides, the percent of berries lost to cranberry fruit rot in Massachusetts and New Jersey ranges from <1% to 15% (Oudemans et al., 1998). In beds where fungicides were not used for disease control, the percent of berries lost to rot ranged from 26% to 100% in New Jersey and <1% to 37% in Wisconsin (Wells-Hansen and McManus, 2017). In contrast to the three other cranberry-growing states the distinctly moderate year-round climate and drier summers in cranberry-growing areas in the Pacific Northwest was proposed to account for historically lower levels of CFRs (Oudemans et al., 1998; Shear et al., 1931). Climate also may affect the species that comprise the cranberry fruit rot complex in the production region along the Pacific coast of Oregon and Washington. A survey of cranberry fruit rot fungi in beds in Grayland, Washington from 1990 to 1992 reported that *Allantophomopsis cytisporea, Botrytis cinerea, Colletotrichum gloeosporioides, Godronia cassandrae, Diaporthe vaccinii*, and *Phyllosticta elongata* were isolated from diseased cranberries (Keates, 1993). To the best of our knowledge, more recent surveys of CFRs in cranberry beds in Oregon or Washington have not been published. To address this knowledge gap, cranberries were collected near harvest from farms in the two main cranberry growing regions in the Pacific Northwest to provide yield estimates, fruit rot incidence, and characterization of the fungi in the cranberry fruit rot complex in this region.

## 2 Materials and methods

### 2.1 Cranberry farms surveyed

Seven commercial cranberry farms (cv. ‘Stevens’) were sampled from 2020 to 2023. Four farms (numbered 1 to 4) were located near Bandon, Oregon (OR) (43°7’8” N 124°24’30” W), on the southern Oregon coast. Three cranberry farms (numbered 5 to 7) were located in Grayland, Washington (WA) (46°48’36” N 124°5’36” W), which also is a coastal town. The distance between the Oregon and Washington field locations was approximately 530 km. The distance from the cranberry farms sampled in either state to the Pacific Ocean was between 0.9 to 4.7 km.

#### 2.1.1 Fungicides applied to cranberry beds

All farms were conventionally-managed except Farm 4 in Bandon, OR, which was certified organic until 2022 and then converted to conventionally-managed in 2023. Fungicide programs are summarized in **Tables 1** and **2**. Oregon growers applied fungicides two or three times during the season. Washington growers, producing cranberries for fresh markets, applied fungicides three to seven times in a season (**Table 2**). Multisite fungicides, which have multiple modes of action and are considered low risk for development of fungicide resistance (Fungicide Resistance Action Committee, 2024), were applied at least once in each farm during the course of this survey, but they were not applied to Farms 1 and 2 every year (**Table 2**). Among the multisite fungicides, only chlorothalonil was applied in Oregon whereas mancozeb was applied in each farm in Washington and chlorothalonil was applied in two of the three Washington farms sampled (**Table 2**). Copper, another multisite biocide used in organic and conventional production, was applied as an early season treatment in four of the seven farms (**Table 2**).

**Table 1:** Fungal isolates from selected Oregon (OR) and Washington (WA) cranberries with field rot symptoms.

				Incidence of isolation^a^									
State	Farm	Year	No. of isolates	Black rots^b^	*Botrytis*	Coleophoma sp.	*Colletotrichum* sp.	*Diaporthe* sp.	*Godronia cassandrae*	*Neofabraea actinidiae*	*Phyllosticta elongata*	*Physalospora vaccinii*	Fungicidal active ingredients applied^c^
OR	1	2022	51	- ^d^	0.02	0.04	0.65	0.04	–	–	–	0.25	Cu,C,C,P
		2023	24	–	–	0.25	–	0.21	–	–	0.25	0.29	P,P,A
	2	2022	52	0.02	0.02	0.33	0.02	0.08	0.04	0.04	0.04	0.42	A,F
		2023	16	–	0.25	–	0.06	0.06	0.13	0.19	–	0.31	F,C
	3	2022	35	0.06	–	0.23	0.17	0.14	–	–	0.09	0.31	P,C
		2023	57	–	–	–	0.02	0.05	–	–	0.04	0.89	P,C
	4	2022	48	0.13	–	0.08	0.27	0.04	0.08	0.25	0.15	–	Cu,Cu,Bs
		2023	29	0.03	–	0.10	0.66	–	0.03	0.03	–	0.14	C,C
WA	5	2021	82	0.20	–	0.51	0.26	0.02	–	–	–	0.01	M,P,M,AD,P,C
		2022	29	0.07	0.10	0.31	0.34	0.10	–	–	–	0.07	Cu,AD,P,C
		2023	51	–	0.10	0.04	0.69	0.06	–	–	0.06	0.06	P,AD,M,P,C
	6	2021	80	0.13	0.01	0.79	0.01	0.04	0.01	0.01	–	–	A,F,A,M
		2022	43	0.02	–	0.26	–	0.05	–	0.09	–	0.58	F,A,M
		2023	60	–	–	0.08	0.38	0.08	0.02	0.10	–	0.33	Cu,A,F,A,M
	7	2021	71	0.37	0.07	0.18	0.06	0.30	0.01	–	–	0.01	M,P,M,AD,P,C
		2022	62	0.23	0.03	0.34	–	0.29	–	0.02	–	0.10	M,P,A,M,P,A,C
		2023	30	–	0.03	–	–	0.20	–	0.03	0.50	0.23	AD,P,M,AD,P,M,C

a Incidence of isolation calculated among the isolates identified from symptomatic cranberries.

b Black rots include *Allantophomopsis cytisporea*, *A. lycopodina*, and *Strasseria geniculata*.

c Active ingredients are listed in order that they were applied with a comma separating each application time. Active ingredients key: A, azoxystrobin; AD, azoxystrobin plus difenoconazole; Bs, *Bacillus subtilis* QST 713; F, fenbuconazole and P, prothioconazole. Multi-site fungicides: C, chlorothalonil; Cu, copper hydroxide and M, mancozeb.

d ‘-’ denotes the fungus was not isolated.

**Table 2:** Fungal isolates from Oregon (OR) and Washington (WA) cranberries stored at 4°C that developed rot symptoms.

					Incidence of isolation^a^									
State	Farm	Year	Storage (weeks)	No. of isolates	Black rots^b^	*Botrytis*	*Coleophoma* sp.	*Colletotrichum* sp.	*Diaporthe* sp.	*Godronia cassandrae*	*Neofabraea actinidiae*	*Phyllosticta elongata*	*Physalospora vaccinii*	Fungicidal active ingredients applied^c^
OR	1	2021^d^	8	22	0.32	-^e^	0.18	0.27	0.18	–	0.05	–	–	Cu,C,C,P
		2022^f^	4	11	0.36	–	0.18	0.18	0.27	–	–	–	–	Cu,C,C,P
		2022	8	8	–	–	0.13	0.50	0.25	–	0.13	–	–	
		2023	6	11	0.36	–	0.36	–	–	–	–	0.27	–	P,P,A
	2	2021	8	70	0.26	–	0.51	0.07	0.07	0.04	0.04	–	–	A,F
		2022	4	6	–	–	0.33	0.17	0.17	0.33	–	–	–	A,F
		2022	8	11	0.27	–	0.18	0.09	0.36	–	0.09	–	–	
		2023	6	13	0.08	–	0.31	0.08	0.08	–	0.15	0.31	–	F,C
	3	2021	8	27	0.15	–	0.37	0.30	0.15	–	0.04	–	–	P,C
		2022	4	0	–	–	–	–	–	–	–	–	–	P,C
		2022	8	5	0.20	–	0.40	–	0.20	–	0.20	–	–	
		2023	6	7	0.14	–	0.29	–	–	–	–	0.29	0.29	P,C
	4	2021^g^	8	128	0.05	–	0.52	0.01	0.02	0.31	0.09	–	–	Cu,Cu,Bs
		2022	4	18	0.17	0.06	–	0.50	–	0.22	0.06	–	–	Cu,Cu,Bs
		2022	8	22	0.05	–	–	0.14	–	0.32	0.41	0.09	–	
		2023	6	20	0.55	0.05	0.05	0.25	–	–	–	–	0.10	C,C
WA	5	2022	4	1	–	–	–	1.00	–	–	–	–	–	Cu,AD,P,C
		2022	8	7	–	–	0.29	0.43	–	–	0.29	–	–	
		2023	6	33	0.03	0.03	0.06	0.64	0.03	–	–	0.21	–	P,AD,M,P,C
	6	2022	4	6	–	0.17	0.33	–	0.33	–	0.17	–	–	F,A,M
		2022	8	5	–	–	0.40	0.20	–	0.20	0.20	–	–	
		2023	6	33	0.42	–	0.03	0.27	0.03	–	0.12	–	0.12	Cu,A,F,A,M
	7	2022	4	10	0.60	0.30	–	–	0.10	–	–	–	–	M,P,A,M,P,A,C
		2022	8	12	0.42	0.08	0.17	–	0.25	–	0.08	–	–	
		2023	6	15	–	–	–	–	0.20	–	0.13	0.67	–	AD,P,M,AD,P,M,C

a Incidence of isolation calculated among the isolates identified from symptomatic cranberries.

b Black rots include *Allantophomopsis cytisporea*, *A. lycopodina*, and *Strasseria geniculata*.

c Active ingredients are listed in order that they were applied with a comma separating each application time. Active ingredients key: A, azoxystrobin; AD, azoxystrobin plus difenoconazole; Bs, *Bacillus subtilis* QST 713; F, fenbuconazole and P, prothioconazole. Multi-site fungicides: C, chlorothalonil; Cu, copper hydroxide and M, mancozeb.

d For Oregon samples in 2021, cranberries with rot symptoms were not removed from samples from beds prior to storage at 4°C

e ‘-’ denotes the fungus was not isolated

f In 2022, asymptomatic cranberries from beds were stored at 4°C and examined for rot symptoms at 4 weeks and 8 weeks. Symptomatic berries were removed at each time point and fungi were cultured from rotted tissues.

g In 2021, three beds from Oregon Farm 4 were sampled.

The FRAC 11 fungicide azoxystrobin was applied as a single fungicide product in two Oregon farms and one Washington farm or as a commercial mixture of azoxystrobin and the FRAC 3 fungicide difenoconazole on beds in two Washington farms (**Table 2**). The FRAC 3 fungicide fenbuconazole (a triazole) was applied on beds in two farms, one located in Oregon and one in Washington, whereas prothioconazole (a FRAC 3 triazolinthione, Fungicide Resistance Action Committee, 2024) was used on four farms with two located in Oregon and two in Washington (**Table 2**).

### 2.2 Sampling of cranberry beds

Annually, cranberries were collected and yield estimates were made near the time of harvest (generally in October; early autumn) with the aid of a fabricated tool consisting of a one-foot diameter (0.09 square meter) metal square. Each of the sampled beds had a rectangular or square footprint covering an area of 0.3 to 1.5 ha. Along three or four of the peripheral edges of a bed, the square metal tool was placed on the surface of the bed. Commercial beds were sampled along the edges to avoid crop damage from walking on the cranberry plants. All berries from the top of the canopy to the bed surface were collected from within the square and placed in a double-layer paper bag. At least two samples were collected from each edge of the bed. Samples along each edge were combined, resulting in at least three replicate samples per bed.

The labeled sample bags were placed in a cooler containing an ice pack and transported to the USDA ARS Horticultural Crops Disease and Pest Management Research Unit laboratory, Corvallis, OR. The sample bags were placed in closed plastic containers and stored at 4°C until berries were sorted and weighed.

### 2.3 Sorting asymptomatic cranberries from those with fruit rot symptoms

#### 2.3.1 Field rots

Cranberries in each replicate sample from a bed were sorted as asymptomatic or berries with symptoms of field rot. The symptomatic berries were sorted further into general categories, such as soft or swollen, discolored (black, brown, pink or yellow), wrinkled, or aborted. For each replicate, the number of berries in each category and the total weight of the berries within each category was recorded. Fungi were isolated from a selection of cranberries with field rot symptoms by the methods described in section **2.4**. The number of asymptomatic berries in each replicate sample and the total weight of the cranberries was recorded.

In 2021, cranberries collected from three Oregon farms were stored at 4°C, but not sorted until 4 to 6 weeks after sampling. The disease incidence data and fungal isolates from the 2021 Oregon samples are presented as storage rots in **Table 2** and likely comprise a mixture of field and storage rots.

#### 2.3.2 Storage rots

In 2022 and 2023, the asymptomatic cranberries in each replicate bed sample were put into 20 cm x 16 cm x 5 cm bags, (Lunchskins, Bethesda, MD), with a limit of 150 berries per bag, placed in a closed plastic bin, and stored in darkness at 4°C for 4 to 8 weeks.

After 4 weeks in 2022, and 6 weeks in 2023, the stored cranberries were sorted as asymptomatic or symptomatic using the same categories for fresh-harvested berries. Cranberries with rot symptoms were removed, counted, and fungi were isolated with the methods described below (section **2.4**). The asymptomatic cranberries were counted and berries within each replicate were weighed, and the asymptomatic cranberries were returned to cold storage. In 2022, after 4 more weeks of storage (8 weeks total), the stored cranberries were sorted, counted, and weighed as before, and cranberries with rot symptoms were processed as described below (section **2.4**).

### 2.4 Isolation of fungi from cranberries with fruit rot symptoms

Selected symptomatic cranberries collected from the field samples (2020 to 2023) or after storage (2022 and 2023) were surface disinfested with 10% commercial bleach (0.6% sodium hypochlorite in deionized water) and 25 µl Tween 20 per L for 10 min, followed by 70% ethanol for 1 min, and then two 1 min rinses with sterile deionized water. Berries were dried in a laminar flow hood (Labconco, Kansas City, MO) until residual water had evaporated. A sample from the leading edge of a lesion (ca. 3 mm^2^) was placed on V8 agar (20% V8 juice, 3 g CaCO_3_ and 15 g Bacto agar (Difco, Becton, Dickinson and Company, Sparks, MD) per L). In cases where entire berries were rotted, the epidermis was removed and a sample of the rotted mesocarp (ca. 3 mm^2^) was placed on V8 agar. In 2023, cranberries with field and storage rot symptoms in Oregon Farms 1, 2 and 4 were sliced longitudinally and the cut surface of the berry was placed on V8 agar. All cultures were incubated at room temperature (ca. 22°C) and outgrowing fungi were transferred at least twice to ensure single-species isolation.

### 2.5 Identification of fruit rot fungi

#### 2.5.1 Colony morphology

Fungal cultures from cranberries were grouped by colony morphology on V8 agar and those with a morphology similar to known CFRs were identified to genus (Wells and McManus, 2013; Polashock et al., 2017; Valentine et al., 2024). Several representative isolates of each colony morphotype were selected each year for ITS 1-4 region sequencing to determine genera and with some fungi, the species.

#### 2.5.2 Analysis of the nuclear internal transcribed spacer region 1-4

Select isolates were cultured on V8 or PDA agar (Difco, Becton, Dickinson and Company, Sparks, MD) for up to 14 days at ambient temperatures and ~50 mg of hyphae was collected and stored at -20°C. Fungal DNA was extracted from the samples by the Core Lab of the Center for Quantitative Life Sciences at Oregon State University, Corvallis, OR with a Thermo KingFisher Flex (Thermo Fischer Scientific, Waltham, MA) and the Omega MagBind Plant DNA DS protocol (Omega Bio-tek, Inc., Norcross, GA).

The nuclear internal transcribed spacer (ITS) region was amplified using primers ITS 1 and ITS 4 (White et al., 1990). Each 25 µl reaction contained of 12.5 µl of AccuStart II PCR ToughMix (Quantabio, Beverly, MA), 0.4 μM of each primer, and approximately 1 to 5 ng of template DNA. PCR conditions were 3 min. at 94°C, 30 cycles: (30 s at 94°C, 30 s at 56°C, 1 min. at 72°C), and final amplification of 10 min. at 72°C. Successful amplification was determined by gel electrophoresis of 5 μl of the reaction on a 1% agarose gel containing ethidium bromide. PCR reaction products were treated with ExoSAP-IT (Thermo Fisher Scientific, Waltham, MA) and sequenced unidirectionally from the ITS 1 primer by the Oregon State University Center for Quantitative Life Sciences, Corvallis, OR. NCBI nucleotide BLAST queries were conducted with each sequence and compared to sequences of type strains of known cranberry fruit rot fungi in GenBank.

### 2.6 Environmental conditions

Weather data were collected by a Washington State monitoring station installed in Grayland, WA and curated by AgWeatherNet (https://weather.wsu.edu/?p=93050) (Washington State University, 2024). Weather data from a monitoring station installed in Bandon, OR, (Latitude 43.09111, Longitude -124.41722) were downloaded from the AgriMet (Pacific Northwest Cooperative Agricultural Weather Network, US Bureau of Reclamation, Columbia-Pacific Northwest Region, website: www.usbr.gov/pn/agrimet/wxdata.html).

### 2.7 Climate data

Climatological data were accessed from https://www.weather.gov/wrh/climate (National Oceanic and Atmospheric Administration, 2024). Locations were selected based on proximity to cranberry production areas: North Bend Southwest Oregon Regional AP, OR; Aberdeen, WA; Wisconsin Rapids, WI; Plymouth Municipal AP, MA; and Hammonton 1 NE, NJ. Monthly average temperature and precipitation were downloaded for 1991 through 2020.

### 2.8 Data analyses

The incidence of cranberry fruit rot was calculated as a ratio of the number of berries with fruit rot symptoms to the total number of berries sampled within a replicate sample. Cranberry yield was estimated based on the total mass (g) of cranberries collected from within 1 ft^2^ metal sampling squares placed on a bed. Yield estimates were converted to Metric Tons per hectare with the following equation:


$$Yield\;\left(\frac{mt}{hectare}\right)=\frac{mass\;sampled\;\left(g\right)}{area\;sampled\;\left(ft^{2}\right)}\frac{107,639\;ft^{2}}{1\;hectare}\frac{1\;mt}{1,000,000\;g}$$

## 3 Results

### 3.1 Environmental conditions during the survey

During the growing season, from bloom through harvest (June through October), Grayland, WA received more rain compared to Bandon, OR in each of the four years of the study (**Tables 3**, **4**). Mean daily solar radiation in Bandon averaged more than 20 MJ/m^2^ during the summer months (June through August) and was generally greater than Grayland, which averaged between 16.5 and 22.6 MJ/m^2^ per day. From June to September each year, air temperatures were generally cool to moderate in both locations, with monthly mean air temperatures below 17°C and low daily temperatures above 0°C (**Tables 3**, **4**).

**Table 3:** Environmental conditions from cranberry bloom to harvest in Bandon, Oregon from 2020 to 2023^a^.

							Precipitation			
					Mean daily solar radiation (MJ/m^2^)	Mean Relative Humidity (%)	Monthly total (mm)	Max daily rainfall (mm)	Mean daily rainfall^b^ (mm)	No. days with rain
		Air temperature (°C)								
Year & Month		Mean	High	Low						
2020	June	13.7 ± 0.2^c^	21.7	5.7	24.1 ± 0.9	87.6 ± 0.9	38.4	9.9	3.2 ± 1.0	12
	July	14.3 ± 0.1	20.4	7.4	25.8 ± 0.6	87.3 ± 0.9	0.8	0.5	0.4 ± 0.1	2
	Aug	15.3 ± 0.2	28.3	6.8	23.7 ± 0.6	85.3 ± 1.4	8.6	4.6	1.7 ± 0.8	5
	Sept	15.6 ± 0.6	36.2	7.1	14.1 ± 1.0	85.6 ± 3.2	57.2	30.7	8.2 ± 4.7	7
	Oct	12.7 ± 0.4	19.6	2.5	12.5 ± 0.5	85.4 ± 2.2	64.3	47.8	4.9 ± 3.6	13
2021	June	13.8 ± 0.2	20.3	4.8	24.3 ± 1.1	89.3 ± 1.2	67.1	35.8	11.2 ± 6.4	6
	July	14.4 ± 0.1	19.9	8.6	24.3 ± 0.8	90.7 ± 0.6	2.0	0.8	0.4 ± 1.0	5
	Aug	14.9 ± 0.2	24.4	7.5	21.7 ± 0.6	87.3 ± 1.0	0.8	0.8	0.8	1
	Sept	14.0 ± 0.3	24.5	4.3	17.7 ± 0.8	87.1 ± 1.6	90.7	44.2	13.0 ± 6.4	7
	Oct	11.6 ± 0.4	22.4	1.6	10.1 ± 0.9	91.0 ± 0.9	157.7	42.2	6.3 ± 1.9	25
2022	June	13.5 ± 0.2	25.6	6.5	22.6 ± 1.3	87.9 ± 1.2	70.1	25.4	5.8 ± 2.0	12
	July	14.8 ± 0.2	22.0	7.6	23.0 ± 1.0	90.3 ± 0.9	1.5	0.8	0.4 ± 0.1	4
	Aug	15.5 ± 0.2	21.2	8.1	20.0 ± 0.9	91.1 ± 1.0	3.8	0.8	0.4 ± 1.0	9
	Sept	14.8 ± 0.2	30.8	7.2	16.0 ± 0.9	90.3 ± 1.5	32.3	10.4	3.2 ± 1.2	10
	Oct	12.4 ± 0.3	27.8	3.5	10.2 ± 0.6	93.1 ± 1.0	42.4	14.5	3.5 ± 1.2	12
2023	June	12.6 ± 0.2	18.5	3.9	24.3 ± 1.0	87.4 ± 1.0	15.0	10.0	2.1 ± 1.3	7
	July	15.1 ± 0.2	21.9	9.7	25.5 ± 0.7	83.8 ± 0.6	1.8	1.8	1.8	1
	Aug	16.4 ± 0.2	23.7	9.7	21.5 ± 0.8	84.1 ± 0.7	6.1	4.6	2.0 ± 1.3	3
	Sept	14.4 ± 0.2	22.6	7.0	16.5 ± 0.9	84.4 ± 0.9	85.3	51.6	10.7 ± 6.2	8
	Oct	12.6 ± 0.5	24.7	1.7	10.7 ± 0.7	85.6 ± 1.5	84.3	24.4	5.6 ± 1.9	15

a Environmental conditions data were downloaded from the AgriMet (Cooperative Agricultural Weather Network, Columbia-Pacific Northwest Region) website (https://www.usbr.gov/pn/agrimet/wxdata.html) from the Bandon, Oregon station (Latitude 43.09111, Longitude -124.41722).

b Mean daily rainfall calculated only among days in a month with measurable precipitation.

c All mean values in the Table are followed by the standard error.

**Table 4:** Environmental conditions from cranberry bloom to harvest in Grayland, Washington from 2020 to 2023^a^.

							Precipitation			
					Mean daily solar radiation (MJ/m^2^)	Mean Relative Humidity (%)	Monthly total (mm)	Max daily rainfall (mm)	Mean daily rainfall^b^ (mm)	No. days with rain
		Air temperature (°C)								
Year & Month		Mean	High	Low						
2020	June	13.6 ± 0.2^c^	24.2	4.4	19.9 ± 1.1	84.0 ± 0.9	66.0	23.6	4.7 ± 1.1	14
	July	14.6 ± 0.2	28.1	5.9	20.7 ± 1.0	85.6 ± 0.9	9.7	2.8	1.0 ± 0.1	10
	Aug	15.5 ± 0.3	30.6	4.8	20.0 ± 0.8	85.8 ± 1.1	42.2	18.3	3.5 ± 0.9	12
	Sept	16.1 ± 0.5	31.8	6.2	11.6 ± 1.1	82.5 ± 3.3	73.9	31.5	6.2 ± 1.6	12
	Oct	11.7 ± 0.5	21.6	-0.6	7.8 ± 0.6	86.5 ± 1.4	127.8	27.9	7.5 ± 1.6	17
2021	June	15.1 ± 0.6	39.8	5.6	22.1 ± 1.2	81.5 ± 1.2	63.2	23.9	4.9 ± 1.3	13
	July	14.5 ± 0.2	20.9	6.7	18.5 ± 1.1	86.1 ± 0.7	5.3	2.0	0.7 ± 0.1	8
	Aug	14.9 ± 0.2	27.6	6.6	16.5 ± 0.9	87.2 ± 0.8	20.8	8.6	1.7 ± 0.4	12
	Sept	13.8 ± 0.3	27.7	4.1	12.3 ± 1.0	86.4 ± 1.1	149.4	40.6	9.3 ± 2.3	16
	Oct	11.0 ± 0.3	17.8	2.2	7.6 ± 0.6	82.4 ± 1.7	253.7	53.3	11.0 ± 2.1	23
2022	June	13.7 ± 0.4	30.5	5.6	17.7 ± 1.4	82.1 ± 1.9	105.7	37.8	5.9 ± 1.8	18
	July	15.3 ± 0.2	27.4	7.5	20.6 ± 0.9	84.6 ± 0.8	9.1	2.8	0.8 ± 0.1	11
	Aug	16.4 ± 0.2	30.7	7.1	16.5 ± 1.0	85.0 ± 1.2	10.2	2.8	0.9 ± 0.1	11
	Sept	15.0 ± 0.3	28.6	5.3	12.3 ± 0.7	82.2 ± 2.2	19.8	15.7	2.5 ± 0.9	8
	Oct	12.4 ± 0.4	27.6	3.4	7.3 ± 0.6	86.8 ± 1.4	142.7	47.2	8.9 ± 2.5	16
2023	June	12.9 ± 0.2	25.0	4.1	22.3 ± 1.1	81.0 ± 1.1	7.9	2.0	0.7 ± 0.1	11
	July	15.1 ± 0.1	26.6	6.9	22.6 ± 1.0	82.5 ± 0.8	20.6	16.5	2.6 ± 1.0	8
	Aug	16.7 ± 0.3	32.6	5.6	18.7 ± 1.1	83.4 ± 1.2	22.9	8.4	3.8 ± 0.6	6
	Sept	14.1 ± 0.3	29.3	2.7	13.5 ± 1.0	84.7 ± 0.8	91.2	23.4	6.1 ± 1.3	15
	Oct	12.3 ± 0.7	27.4	-2.6	7.3 ± 0.6	81.4 ± 1.9	152.4	30.2	10.2 ± 1.9	15

a Environmental conditions data were downloaded from the AgWeatherNet (Washington State University) website (https://weather.wsu.edu/) from the Grayland, Washington station (Latitude 46.78720, Longitude -124.07935).

b Mean daily rainfall calculated only among days in a month with measurable precipitation.

c All mean values in the Table are followed by the standard error.

One notable weather event during the study was an unusual ‘heat dome’ in the Pacific Northwest in late June of 2021. During the ‘heat dome,’ daily maximum temperatures in Grayland, WA were 28°C (June 26), 40°C (June 27), and 35°C (June 28); these temperatures during bloom and berry set were 20°C higher than the 1991 to 2020 average maximum temperature of 18.2°C and 20.1°C in the area for June and July, respectively (Washington State University, 2024; National Oceanic and Atmospheric Administration, 2024). Bandon, located on the southern Oregon coast did not experience the ‘heat dome’ and the maximum high temperature during this period was 20°C (AgriMet, accessed May 2024).

### 3.2 Yield and rot incidence

**Figure 1:**
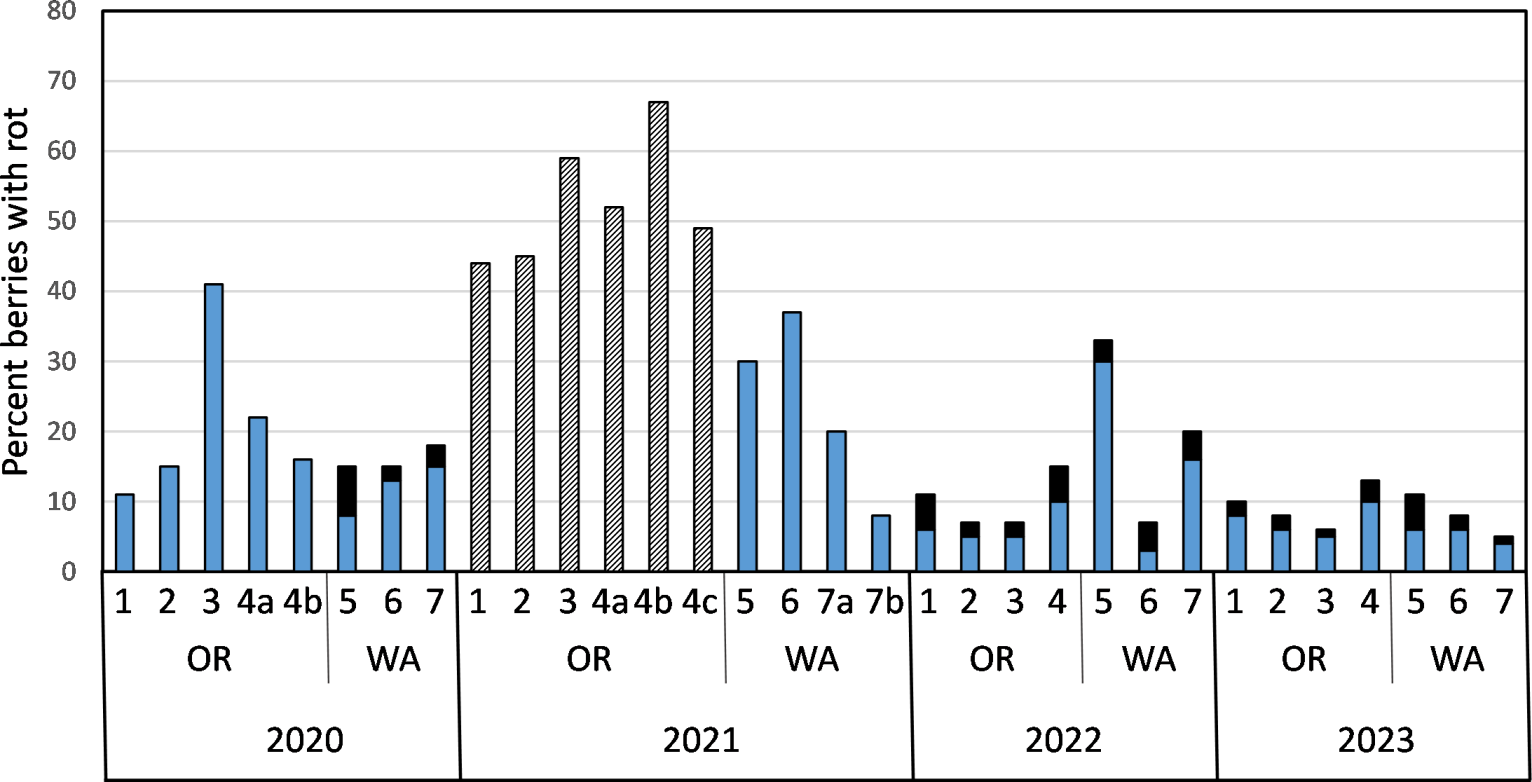
Percent cranberries with fruit rot symptoms in samples collected from beds on farms in Oregon (OR) and Washington (WA) from 2020 to 2023 (blue bars, field rots). Percent of asymptomatic cranberries from field samples that subsequently developed fruit rot symptoms during storage at 4°C for four to eight weeks (solid black bars, storage rot). Black and white striped bars represents total percent fruit rot of cranberries that were collected from Oregon beds in 2021. These field samples consisted of sound and diseased berries that were bulk-stored in paper bags at 4°C for several weeks prior to sorting and assessing the percent total rot (field rot and storage rot).

Over the course of four years of sampling seven farms, 43,260 berries were sorted, weighed, and in 2022 and 2023, all asymptomatic berries were stored at 4°C before sorting again to assess storage rot. Estimates of both yield and rot incidence varied, sometimes greatly, over the years within beds, as well as between states and among farms (**Table 5**). In general, yield per acre was higher in Oregon beds, though it was notably lower in the organically managed farm (#4 from 2020 through 2022) than the three conventionally managed Oregon farms (#1 through 3). Field rot incidence, though variable, was similar among Oregon and Washington beds each year except in 2021 (**Figure 1**, **Table 5**). In 2021, a delay in sorting Oregon cranberries prevented an assessment of field rot incidence. The total percent of cranberries with rot symptoms in Oregon 2021 samples stored at 4°C for several weeks prior to sorting was two to four-fold greater than total rot observed in Oregon cranberries sampled in 2020 and five to 12-fold greater than total rot observed in 2022 and 2023 (**Figure 1**). In Washington state in 2021, the total percent rot in Farm 6 was two to five-fold higher than in the other years of the survey (**Figure 1**).

**Table 5:** Estimated crop yield and incidence of cranberry fruit rot in beds and after storage at 4°C in commercial cranberry beds in Oregon (OR) and Washington (WA) from 2020 to 2023.

State	Farm	Year	Total berry count^a^	Field Rot incidence^b^	Storage^c^ (weeks)	Storage Rot Incidence^d^	Total Yield (mt/ha)^e^
OR	1	2020	1627	0.11 ± 0.03	nd^f^	nd	38.3 ± 0.9
		2021^g^	1318	nd	8	0.44 ± .09	30.0 ± 3.4
		2022	1508	0.06 ± 0.01	8	0.05 ± 0.01	26.5 ± 3.6
		2023	1437	0.08 ± 0.01	6	0.02 ± 0.01	31.5 ± 4.4
	2	2020	1203	0.15 ± 0.03	nd	nd	29.1 ± 2.5
		2021	1660	nd	8	0.45 ± 0.06	36.8 ± 8.4
		2022	1513	0.05 ± 0.01	8	0.02 ± 0.01	36.0 ± 2.3
		2023	1171	0.06 ± 0.01	6	0.02 ± 0.003	27.1 ± 3.5
	3	2020	1165	0.41 ± 0.14	nd	nd	33.2 ± 4.4
		2021†^h^	824	nd	5	0.59 ± 0.05	17.4 ± 4.3
		2022	1626	0.05 ± 0.01	8	0.02 ± 0.003	29.9 ± 4.1
		2023	1298	0.05 ± 0.01	6	0.01 ± 0.002	24.1 ± 3.0
	4	2020†-a^i^	1678	0.22 ± 0.03	nd	nd	30.4 ± 1.3
		2020-b	1301	0.16 ± 0.02	nd	nd	15.2 ± 2.8
		2021-a	1142	nd	2	0.52 ± 0.07	21 ± 2.6
		2021†-b	1435	nd	2	0.67 ± 0.13	26.8 ± 1.8
		2021-c	809	nd	2	0.49 ± 0.02	27.0 ± 0.6
		2022-a	1828	0.10 ± 0.01	8	0.05 ± 0.01	5.7 ± 1.4
		2023-d	903	0.10 ± 0.02	6	0.03 ± 0.01	21.4 ± 4.6
WA	5	2020 ^j^	722	0.08	6	0.07	24.7
		2021	1575	0.30 ± 0.05	nd	nd	30.3 ± 4.7
		2022†	1465	0.30 ± 0.05	8	0.03 ± 0.01	18.1 ± 3.1
		2023	1136	0.06 ± 0.02	6	0.05 ± 0.01	19.1 ± 4.4
	6	2020	916	0.13	6	0.02	17.3
		2021	1426	0.37 ± 0.05	nd	nd	21.5 ± 4.0
		2022	1985	0.03 ± 0.01	8	0.04 ± 0.01	14.0 ± 1.1
		2023	1306	0.06 ± 0.01	6	0.02 ± 0.01	21.0 ± 1.2
	7	2020-a	979	0.15	6	0.03	14.5
		2021-a	1111	0.20 ± 0.13	nd	nd	15.1 ± 1.9
		2021-b	1432	0.08 ± 0.02	nd	nd	16.6 ± 1.8
		2022-c	2366	0.16 ± 0.06	8	0.04 ± 0.01	12.6 ± 4.1
		2023-c	1138	0.04 ± 0.01	6	0.01 ± 0.003	19.3 ± 3.0

a Total berry count is sum of symptomatic and asymptomatic cranberries collected from a bed.

b Field rot incidence is the mean proportion of symptomatic berries to asymptomatic berries in three replicate samples from a bed and the standard error of the mean.

c Storage indicates the number of weeks that asymptomatic berries of each replicate sample were held at 4°C in a sealed plastic box before determining the number of berries that developed symptoms.

d Storage rot incidence is the mean proportion of symptomatic berries to asymptomatic berries in three replicate samples from a bed and the standard error of the mean after storage at 4°C.

e Total yield (metric tons per hectare) was estimated from the combined weight of all cranberries collected from a bed divided by the total area sampled, which was determined with a 1 ft square sampling tool.

f nd denotes not determined.

g All Oregon 2021 samples were stored at 4°C for various lengths of time before cranberries were examined for symptoms. The rot incidence data is presented as storage rot, which for these beds is a combination of field rot symptoms and symptoms that developed during storage.

h The dagger symbol (†) indicates that the cranberry beds were harvested prior to sample collection.

i A letter following the year shows a rotation of beds sampled on Oregon Farm 4 and Washington Farm 7 due to bed renovation. The year followed by a common letter indicates that the same bed was sampled in different years.

j Three replicate samples were collected from beds in Washington state in 2020 and combined into one sample container at the field site. Rot incidence was calculated from the number of cranberries with symptoms and the total number of berries.

### 3.3 Identification of CFRs and non-CFRs

**Figure 2:**
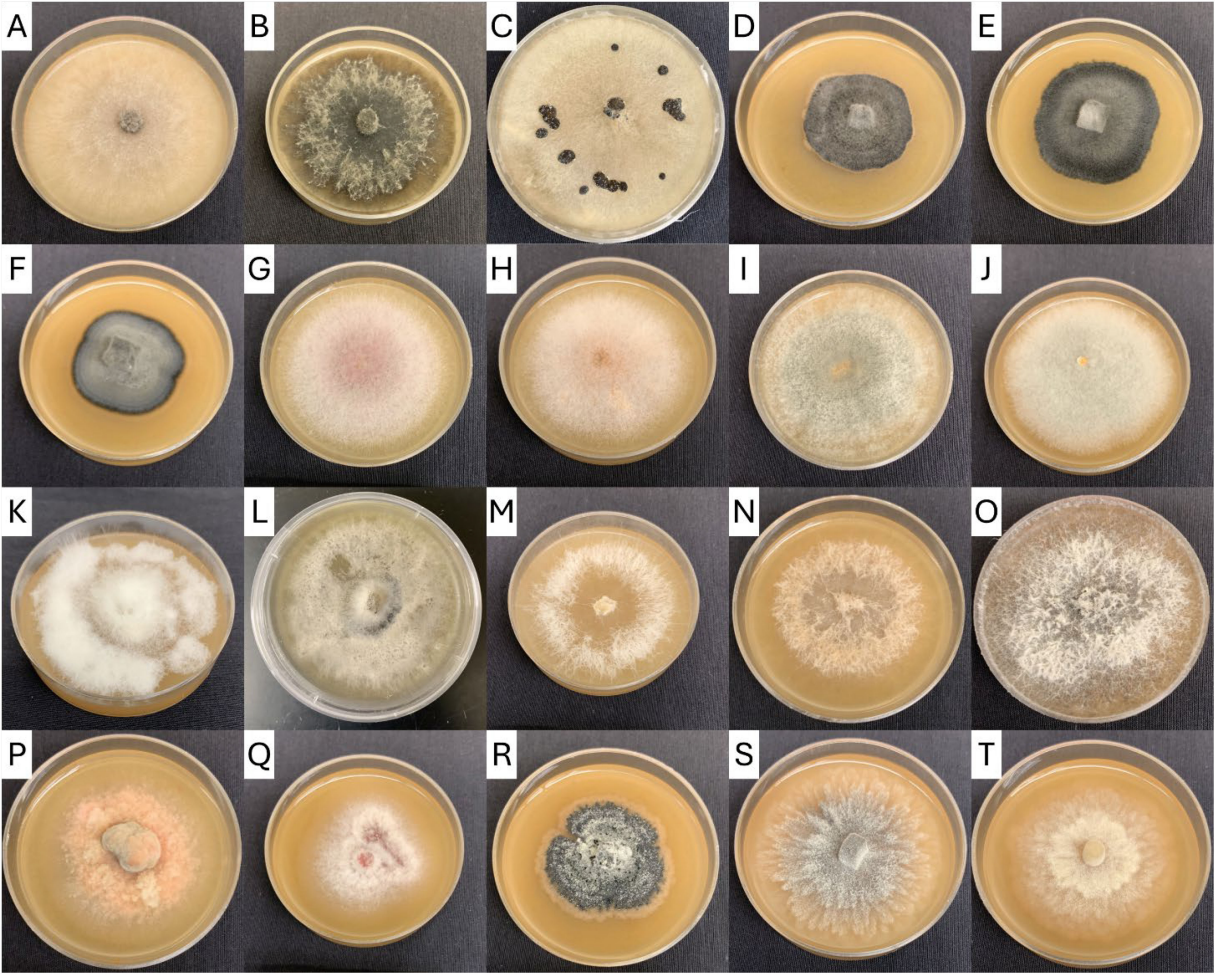
Cranberry fruit rot fungi isolated from Oregon and Washington beds and cultured on V8 agar. **(A)** *Allantophomopsis cytisporea*, **(B)** *Allantophomopsis lycopodina*, **(C)** *Botrytis* sp., **(D–F)** *Coleophoma* sp., **(G–J)** *Colletotrichum acutatum* sp., **(K, L)** *Colletotrichum gloeosporioides* sp., **(M–O)** *Diaporthe* sp., **(P)** *Godronia cassandrae*, **(Q)** *Neofabraea actinidiae*, **(R)** *Phyllosticta elongata*, **(S)** *Physalospora vaccinii* (dark strain), **(T)** *Physalospora vaccinii* (light strain).

From 2021 through 2023, over 1500 fungi were isolated from cranberries and identified at least to genera. The incidence of identified CFRs from field-rotted berries are summarized in **Table 1** (identity of CFRs isolated from field rotted berries). Similarly, the incidence of recovery of various CFR from stored cranberries are presented in **Table 2** (identity of CFRs isolated from storage-rotted cranberries). Examples of the colony morphology of isolates grown on V8 agar are shown in **Figure 2**. The identity of each isolate was verified with ITS sequence analysis.

In **Tables 1** and **2**, *Colletotrichum* isolates were only identified to the complex level (*acutatum* or *gloeosporioides*), not necessarily to species, and are listed as “*Colletotrichum.” Diaporthe* sp. were not identified to species using the ITS region and are listed as “*Diaporthe.”* In addition to the cranberry ripe rot pathogen *Coloephoma cylindrospora*, other *Coleophoma* species were isolated frequently. It was not determined if the other *Coloephoma* species are pathogenic to cranberries, so all *Coloephoma* species were grouped and referred to as “*Coleophoma.*” Black rots, including *A. lycopodina*, *A. cytisporea*, and *S. geniculata*, were not identified to species using the ITS region, and are listed as “black rots.”

During the survey, we isolated nearly all previously reported CFRs in the Pacific Northwest except for *M. oxycocci*, which almost 100 years ago “In Wisconsin and on the Pacific coast [*M. oxycocci* was] practically the only fungus that has been found to cause field rot … [but] never exceeded a fraction of 1 per cent of the total crop” (Shear et al., 1931). Historically, *Phyllosticta vaccinii* also was reported to cause CFR in the Pacific Northwest, but this fungus was later separated into two species, *Phyllosticta vaccinii* and *Phyllosticta elongata* (Weidemann et al., 1982). *Phyllosticta elongata* was isolated as a CFR in this survey, but not *Phyllosticta vaccinii*. Due to historic taxonomic ambiguity, it is unclear if *Phyllosticta vaccinii* was isolated previously from cranberries in the Pacific Northwest or if the isolates were *Phyllosticta elongata*. *N. actinidiae*, recently reported as a cranberry fruit rot (Valentine et al., 2024), was isolated from beds in both states. Previously, *N. actinidiae* was isolated from cranberry woody tissue in British Columbia, Canada (Sabaratnam et al., 2009), but not as a fruit rot. *N. actinidiae* was most frequently isolated from cranberries stored at 4°C, but the pathogen also was frequently isolated from field rotted cranberries (**Tables 1**, **2**).

Genera of fungi not known to cause cranberry fruit rot were isolated frequently and included *Pestalotiopsis*, *Epicoccum*, *Cadophora*, *Cladosporium, Penicillium, Aureobasidium*, *Lachnum*, and *Alternaria* (data not shown). The cranberry leaf pathogen *Cladosporium oxycocci* (Polashock et al., 2017), was most often isolated when plating half-berries in 2023, as opposed to smaller samples of rotted berry tissue as done in the prior years. Several non-CFRs could not be identified to genus using only the ITS 1-4 region and would require further research to determine identity.

### 3.4 Persistence of rot pathogens in beds

Despite the variability of rot incidences and causal agents, certain trends were apparent over time. Several cranberry fruit rot pathogens were isolated at least once in each farm in Oregon and Washington, as either a field rot, storage rot, or both (**Tables 1**, **2**). These included black rots (*A. cytisporea*, *A. lycopodina*, and *S. geniculata*), *Coleophoma*, *Colletotrichum*, *Diaporthe*, *N. actinidiae*, *Physalospora vaccinii*, and *Phyllosticta elongata*. The remaining CFR pathogens often were isolated from the same beds over multiple years. For example, *G. cassandrae* was isolated multiple times from Farms 2, 4, and 6, but only once from Farm 7 in 2021 (**Table 1**). There was no clear or consistent correlation between fungicides applied during the growing season and CFR pathogens isolated (**Tables 1**, **2**).

### 3.5 Climate differences in the five main cranberry producing states

**Figure 3:**
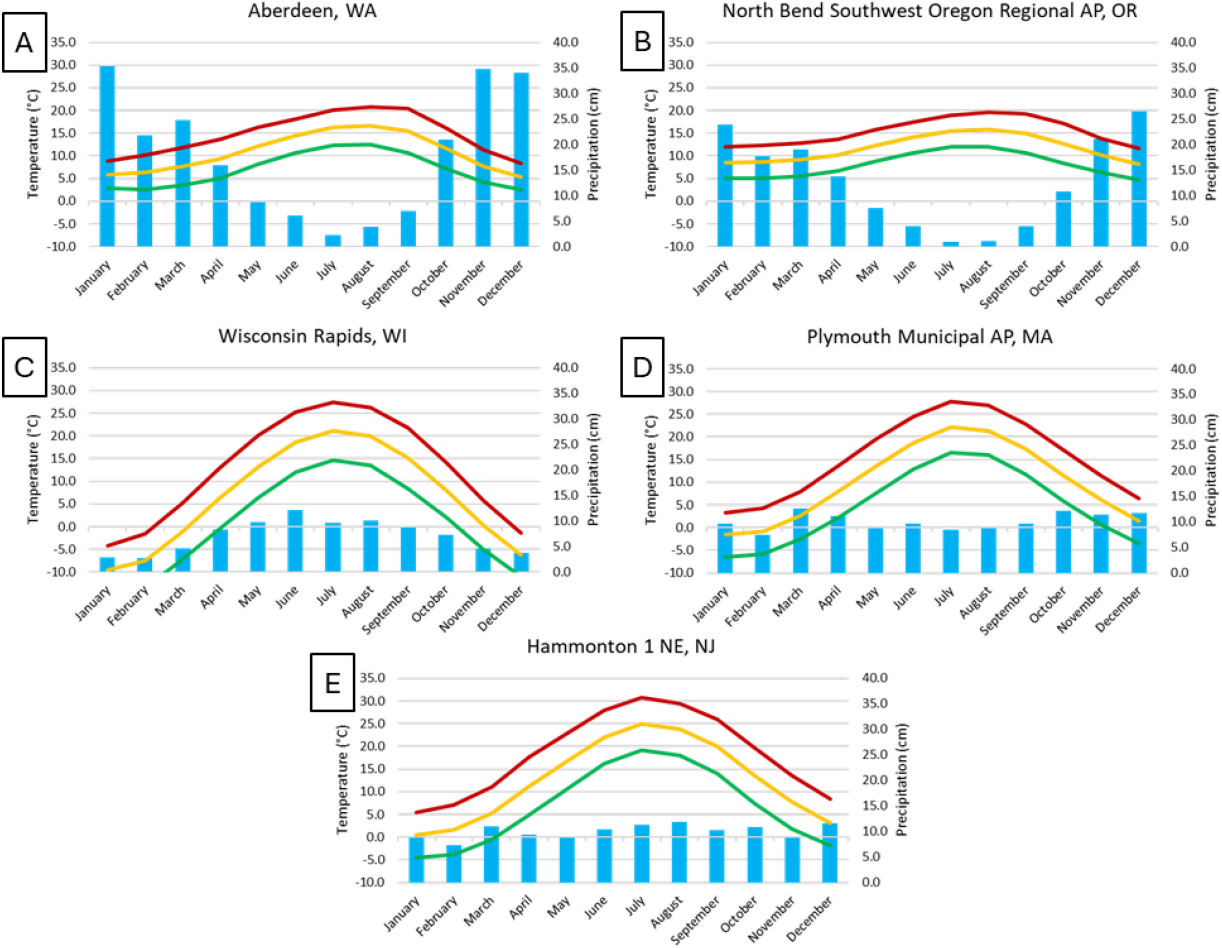
Climate graphs of monthly mean maximum (red line), minimum (green line), average temperature (yellow line) (°C), and mean total precipitation (blue bars) (cm), for 1991-2020, using data from National Oceanic and Atmospheric Administration (2024). Locations of stations: **(A)** Aberdeen, Washington (Northeast of Grayland, Washington), **(B)** North Bend Southwest Oregon Regional AP, Oregon (North of Bandon, Oregon), **(C)** Wisconsin Rapids, Wisconsin, **(D)** Plymouth Municipal AP, Massachusetts, **(E)** Hammonton 1 NE, New Jersey. Cranberry plants generally bloom in May to June in each state, except Wisconsin, when bloom occurs later, in mid-June to July. Cranberries are harvested from late-September into November across the production regions.

There are notable differences between the climate in Oregon and Washington compared to the other three cranberry producing states (**Figure 3**). Overall precipitation was higher on average in the Pacific Northwest regions, especially in Washington, but the majority of rainfall occurred in mid-autumn to late spring (October to May), and the rest of the year (June to September, the main growing season for cranberries) was drier than monthly averages in the other three cranberry growing regions, where precipitation was relatively constant over the year. In Wisconsin, however, winter months were drier, averaging less than 5 cm precipitation, and summer months averaged more than 10 cm. Temperatures in the Pacific Northwest growing regions were mild, with mean highs below 21°C in the summer, and mean lows generally above freezing in the winter. For the Wisconsin, Massachusetts, and New Jersey cranberry-growing regions, mean high temperatures often exceeded 25°C in the summer months and mean low temperatures below 0°C were recorded during the winter months.

## 4 Discussion

### 4.1 The heterogeneity of cranberry bogs

A common pattern of cranberry yield and cranberry rot incidence over the duration of this study was the patchy distribution of rots within beds. This was noted in previous studies with regard to rot incidence and CFR types (McManus et al., 2003; Wells et al., 2014). This was apparent with the large variance in average rot incidence or yield calculations from several of the farms over time (**Table 1**). Variation among the pathogens isolated from different locations within a bed also was observed. For example, certain rots, like *Colletotrichum*, were isolated frequently from samples from one edge of a bed, but not from samples collected from the other edges of the same bed (data not shown). The sheer number of cranberries that need to be sampled and cultured to determine the prevalence of the various fruit rot fungi in a bed accurately is a daunting task. Recent advances in tools, like multiplex PCR to identify fungal pathogens in cranberries, may support large-scale surveys of the cranberry fruit rot pathogen complex (Conti et al., 2022). Pathogen diversity at the genus and species level and the spatial distribution of pathogen within and among cranberry beds underlines the complexity of studying cranberry fruit rots. Nonetheless, several distinct and important trends were observed which may shed light on factors underlying these complex relationships.

### 4.2 Environment and fungicide use may drive rot incidence and CFR composition

We speculate that environmental conditions in Oregon and Washington shape the CFR fungal community composition in the Pacific Northwest region. Mild winter and summer temperatures in Grayland WA, and especially Bandon, OR, as well as low precipitation during the growing season (**Tables 3**, **4**), likely affects expression of rots as well as species composition. The question is, by what ways does this occur? *C. acutatum* species grow better at lower temperatures than *C. gloeosporioides* species (Dowling et al., 2020), and *C. acutatum* accounted for most of the *Colletotrichum* isolates in 2022 and 2023. The incidence of *Neofabraea*, another species with a lower optimum growth temperature and a storage pathogen on kiwifruit and several other crops (Bogo et al., 2018; Tyson et al., 2019), might be recovered as a cranberry fruit rot in beds in Oregon and Washington because of the cool summer temperatures that are not duplicated in cranberry growing regions outside the Pacific Northwest. Similarly, the ‘Black rot’ complex is primarily described as fungi causing cold-storage rots of cranberries (Polashock et al., 2017), but these pathogens were isolated from cranberries that developed field-rot symptoms in beds in Oregon and Washington, suggesting that the temperatures were sufficiently low to support the expression of black rot symptoms in the field.

Field rot incidence was relatively low each year in all beds, except during 2020 and 2021. Field rot was generally higher in the organically-managed Oregon beds (**Table 5**, Farm #4, 2020 through 2022) in comparison to the conventionally-managed Oregon beds (Farms 1 to 3). Among the Washington beds, the highest incidence of field rot occurred in 2021, where cranberry fruit rot incidence was between 0.08 to 0.37 (**Table 5**, Farms 5 to 7). The increased incidence of field rot in Grayland in 2021 may have been influenced by sudden heat stress (temperatures around 40°C for three days) in late June, 2021 during bloom and pinhead stage. We speculate that the sudden high temperatures damaged developing carposphere tissues and increased the incidence of infection. We noted that the incidence of isolation of black rot fungi and *Coleophoma* sp. was greater from field rotted berries in 2021 Washington beds than the other years (**Table 1**). This disproportionate representation of field rots in 2021 Washington beds might have occurred via preferential infection of flowers by spores of the CFRs. Additionally, fungicides were applied prior to or during the heat stress event, but we speculate that watering to reduce heat damage may have reduced the concentration of fungicide residues on plants surfaces and disease management efficacy.

Multi-site fungicides, like chlorothalonil and mancozeb, have been highly effective in managing cranberry fruit rots (Wells et al., 2014). However, the U.S. Environmental Protection Agency has listed both compounds as potential carcinogens (Environmental Protection Agency, 2024). This, and the registration of specific single-site fungicides for use in cranberry production, has shifted management of cranberry fruit rot from multisite chemistries to single-site fungicides, and to some extent reduced use of multi-site fungicides.

The single site fungicides used during the survey in Oregon and Washington belong to FRAC groups 3 and 11, which are often considered safer to humans and other mammals than multisite materials. Unfortunately, some of the site-specific fungicides may not be effective against specific fungi within the CFR complex due to intrinsic tolerance to the active ingredient (Fungicide Resistance Action Committee, 2024). Additionally, there is an increased risk of resistance developing to single-site fungicides compared to multi-site fungicides (Fungicide Resistance Action Committee, 2024). At this time, the sensitivities of the CFR fungi to various single-site fungicides has not been fully demonstrated. Nonetheless, the incidence of isolation of *Godronia* and *Neofabraea* among cranberries with fruit rot symptoms was low in samples from beds treated with prothioconazole, compared to beds treated with fenbuconazole (**Tables 1** and **2**). This field data may indicate that *Godronia* and *Neofabraea* exhibit greater field sensitivity to prothioconazole (FRAC 3 triazolinthione fungicide) than to fenbuconazole (FRAC 3, triazole fungicide) (Fungicide Resistance Action Committee, 2024). These observations align with recent fungicide sensitivity assays by Wood et al. (2023). They demonstrated higher efficacy of prothioconazole compared to fenbuconazole to reduce mycelial growth and spore germination among CFRs and that *G. cassandrae* isolates were sensitive to both azole chemistries (Wood et al., 2023). Characterizing the CFR complex members in a bed and their intrinsic tolerance or sensitivity to various fungicides may lead to tailored CFR management programs.

### 4.3 A Glimpse into the cranberry microbiome and potential for additional CFRs

Several fungi not reported to cause cranberry fruit rots were isolated during this study and have been isolated from cranberries in other regions of North America (Sabaratnam et al., 2009; Wood et al., 2023) and warrant further study on their ecological role in cranberry beds. Some fungi, like *Alternaria*, *Cadophora*, and *Penicillium*, may be capable of causing a cranberry fruit rot but their ability to cause disease on cranberries has not been determined. In highbush blueberry (*Vaccinium corymbosum*), another *Vaccinium* sp. that shares many important pathogens with cranberry, *Alternaria tenuissima* can cause leaf spot and fruit rot (Polashock et al., 2017). *Cadophora luteo-olivacea* can cause a postharvest rot in kiwifruit (*Actinidia deliciosa*) (Auger et al., 2019), while *Penicillium expansum* can cause postharvest rot in pome fruit (Luciano-Rosario et al., 2020).

Other fungi may grow on plant surfaces as a non-pathogenic endophyte or as an epiphyte. The geographic isolation and climate differences may be important drivers of non-characterized fungi isolated from rotted cranberries. Some fungi, such as A*ureobasidium*, may have biocontrol potential, while *Lachnum* sp. isolated from cranberry plants in Quebec, Canada, have shown biocontrol activity against some known CFRs (Freimoser et al., 2019; Salhi et al., 2022). These genera were not isolated from some cranberry beds, perhaps related to fungicide use or lack of inoculum sources. We speculate that understanding the effects of fungicides on species which may compete with known CFRs, but do not cause fruit rot, may be important in determining how to improve conditions for species with putative biocontrol properties.

### 4.4 Constraints, biases, and alternative methods

Sampling of commercial cranberry beds had several constraints and potential biases. Sampling only occurred at the edges of the beds to avoid crop damage (except for farm 6 in 2022, in which dry-harvest was halfway completed, so sampling occurred on a portion of the bed), and in some cases, because of steep inclines on the sides of one bed (Farm 1), not every side of the bed was sampled. In Farm 3 (2020), and Farm 5 (2022), sampling occurred after harvest, around sprinklers and the extreme edges of the beds. All of these may have contributed to biases in CFR composition, as well as yield and rot incidence estimates. The 2021 Oregon cranberry fruit rot incidences from each field were higher than in the other years of the study (0.44 to 0.67, **Table 5**, **Figure 1**). The environmental conditions and fungicide use were similar to those in other years with lower fruit rot incidences (**Tables 3**, **5**, and **2**). However, unlike samples from other years in Oregon, the 2021 cranberry samples were stored at 4°C for 4 to 6 weeks prior to sorting and determination of rot incidence. We speculate that during storage, CFR fungi may have spread from diseased berries to sound berries collected from beds, resulting in a greater incidence of rot compared to samples from beds where symptomatic cranberries were removed prior to storage. While we cannot determine what the incidence of field or storage rot for the 2021 samples from Oregon would have been if the berries had been sorted prior to storage, these data support the importance of removing symptomatic berries on processing lines to reduce storage rots, especially for fresh-market cranberries.

A common protocol for isolating fungi from surface-disinfested cranberries is to cut the entire berry longitudinally, and place the cut side of the berry on the surface of solidified culture media, such as V8, corn meal, or potato dextrose agar (McManus et al., 2003; Olatinwo et al., 2003; Polashock et al., 2009; Wood et al., 2023). Our method to isolate CFRs often involved placing a sample of the lesion margin or internal mesocarp tissue onto media, which frequently resulted in isolation of a single fungus from a cranberry with rot symptoms. We suspect that some pathogens may be overrepresented as fruit rots when plating half of a berry. Multiple fungi may infect the same fruit, and plating half of the cranberry may increase the likelihood of isolating fungi that have not caused rot in that cranberry, even if the fungus is a known CFR. Additionally, inclusion of a more advanced rotted section of berry may increase the likelihood of isolating opportunistic fungi that can more readily grow on already rotted berry flesh, as opposed to asymptomatic cranberries. Conversely, our method of sampling symptomatic tissues may be prone to not culturing pathogenic fungi residing in berries as a latent infection.

This study, despite its constraints and potential biases, provides a glimpse into the CFR complex in commercial cranberry beds in two main growing regions of the Pacific Northwest. The main CFRs isolated from Oregon and Washington included *Colletotrichum* (largely from the *acutatum* complex), *Physalospora vaccinii*, and *Coleophoma* sp. From recent studies in Quebec and British Columbia, Canada, *G. cassandrae* and *Phyllosticta elongata* were the main species observed, respectively (Conti et al., 2022; Wood et al., 2023). In our study, these fungi were found at much lower incidences overall. In Quebec and British Columbia, *Physalospora vaccinii* and *C. empetri* also were found at relatively greater incidences than most other CFRs in those studies. In recent studies from New Jersey and Wisconsin, *Colletotrichum* was isolated frequently in both states, though *C. gloeosporioides* was more prevalent in New Jersey than Wisconsin and *C. empetri* was reported to be more prevalent in Wisconsin (Polashock et al., 2009; Wells-Hansen and McManus, 2017). *Physalospora vaccinii* was isolated from most beds sampled in Oregon and Washington. These studies all suggest variations in main CFR types among growing regions, though certain species and complexes, like *Physalospora vaccinii*, and *Colletotrichum* sp., tend to be highly represented, if not dominant, regardless of region. Further elucidation of interactions between CFR fungi, rot incidence, environmental conditions, and fungicides will be useful to develop and deploy more localized management strategies. This includes additional studies on the sensitivity of various CFR fungi to fungicides, as well as the effect of climate and environment on the expression of cranberry fruit rots. The sheer number of potential CFR fungi in cranberry beds makes this a daunting but nevertheless valuable undertaking.

## Data Availability

The original contributions presented in the study are included in the article/supplementary material. Further inquiries can be directed to the corresponding author.

## References

[B1] Auger J. Pérez I. Osorio-Navarro C. Esterio M. (2019). First report of *Cadophora luteo-olivacea* causing side rot on kiwifruit in Chile. Plant Dis. 102, 680. doi: 10.1094/PDIS-09-17-1349-PDN

[B2] Bogo A. Comparin C. C. Valdebenito Sanhueza R. M. Ritschel P. Casa R. T. Silva F. N.. (2018). Characterization of *Neofabraea actinidiae* and *N. brasiliensis* as causal agents of apple bull’s-eye rot in southern Brazil. Can. J. Plant Pathol. 40:2, 229–237. doi: 10.1080/07060661.2017.1421588

[B3] Conti M. Cinget B. Labbé C. Asselin Y. Bélanger R. R. (2022). New insights into the fungal diversity of cranberry fruit rot in Québec farms through a large-scale molecular analysis. Plant Dis. 106, 215–222. doi: 10.1094/PDIS-06-21-1163-RE 34515508

[B4] Dowling M. Peres N. Villani S. Schnabel G. (2020). Managing Colletotrichum on fruit crops: A “complex. challenge. Plant Dis. 104, 2301–2316. doi: 10.1094/PDIS-11-19-2378-FE 32689886

[B5] Environmental Protection Agency. (2024). Available online at: https://www.epa.gov (Accessed June 12, 2024).

[B6] Freimoser F. M. Rueda-Mejia M. P. Tilocca B. Migheli Q. (2019). Biocontrol yeasts: mechanisms and applications. World J. Microbiol. Biotechnol. 35, 154. doi: 10.1007/s11274-019-2728-4 31576429 PMC6773674

[B7] Fungicide Resistance Action Committee. (2024). Fungicide resistance management. Available online at: https://www.frac.info/ (Accessed May 3, 2024).

[B8] Keates S. E. (1993). Endophytic fungi associated with *Vaccinium macrocarpon* Ait. (Cranberry) in Washington state and the effect of fungicide treatments on their occurrence. Washington State University, Pullman (WA.

[B9] Luciano-Rosario D. Keller N. P. Jurick W. M. II (2020). *Penicillium expansum*: biology, omics, and management tools for a global postharvest pathogen causing blue mould of pome fruit. Mol. Plant Pathol. 21, 1391–1404. doi: 10.1111/mpp.12990 32969130 PMC7548999

[B10] McManus P. S. Caldwell R. W. Voland R. P. Best V. M. (2003). Evaluation of sampling strategies for determining incidence of cranberry fruit rot and fruit rot fungi. Plant Dis. 87, 585–590. doi: 10.1094/PDIS.2003.87.5.585 30812963

[B11] National Oceanic and Atmospheric Administration. (2024). Climate. Available online at: https://www.weather.gov/wrh/climate (Accessed May 1, 2024).

[B12] Olatinwo R. O. Hanson E. J. Schilder A. M. C. (2003). A first assessment of the cranberry fruit rot complex in Michigan. Plant Dis. 87, 550–556. doi: 10.1094/PDIS.2003.87.5.550 30812957

[B13] Oudemans P. V. Caruso F. L. Stretch A. W. (1998). Cranberry fruit rot in the Northeast: A complex disease. Plant Dis. 82, 1175–1283. doi: 10.1094/PDIS.1998.82.11.1176 30845403

[B14] Polashock J. J. Caruso F. L. Averill A. L. Schilder A. C. (2017). Compendium of Blueberry, Cranberry, and Lingonberry Diseases and Pests (St. Paul: APS Press).

[B15] Polashock J. J. Caruso F. L. Oudemans P. V. McManus P. S. Crouch J. A. (2009). The North American cranberry fruit rot fungal community: a systematic overview using morphological and phylogenetic affinities. Plant Pathol. 58, 1116–1127. doi: 10.1111/j.1365-3059.2009.02120.x

[B16] Sabaratnam S. Dicarlo A. Fitzpatrick S. Forge T. (2009). Cranberry dieback disorder: a new and emerging threat to cranberry production in British Columbia. Acta Hortic. 810, 417–424. doi: 10.17660/ActaHortic.2009.810.54

[B17] Salhi L. N. Villalobos P. B. Forget L. Burger G. Lang B. F. (2022). Endosymbionts in cranberry: Diversity, effect on plant growth, and pathogen biocontrol. Plants People Planet. 4, 511–522. doi: 10.1002/ppp3.10290

[B18] Shear C. L. Stevens N. E. Bain H. F. (1931). Fungous Diseases of the Cultivated Cranberry. Technical Bulletin No. 258 (Washington, D.C: United States Department of Agriculture).

[B19] Strik B. Bristow P. Broaddus A. Davenport J. DeFrancesco J. T. English M.. (2002). Cranberry production in the Pacific Northwest. Pacific Northwest Extension 247, 55–59.

[B20] Tyson J. L. Manning M. A. Everett K. R. Fullerton R. A. (2019). *Neofabraea actinidiae* in New Zealand kiwifruit orchards: current status and knowledge gaps. New Z. Plant Protection. 72, 75–82. doi: 10.30843/nzpp.2019.72

[B21] United States Department of Agriculture, National Agricultural Statistics Service. (2019). Noncitrus fruits and nuts 2018 Summary. 37–38

[B22] United States Department of Agriculture, National Agricultural Statistics Service. (2024). Noncitrus fruits and nuts 2023 Summary. 34

[B23] Valentine D. C. Shaffer B. T. McGhee G. C. Bouska C. Stockwell V. (2024). First report of *Neofabraea actinidiae* causing a cranberry fruit rot in Oregon. Plant Dis. 108, 1405. doi: 10.1094/PDIS-11-23-2526-PDN

[B24] Washington State University. (2024). (AgWeatherNet). Available online at: https://weather.wsu.edu/?p=93050 (Accessed May 1, 2024).

[B25] Weidemann G. J. Boone D. M. Burdsall H, H. (1982). Taxonomy of *Phyllostica vaccinii* (*Coelomycetes*) and a new name for the true anamorph of *Botryosphaeria vaccinii* (*Dothideales*, *Dothioraceae*). Mycologia 74; 1, 59–65. doi: 10.1080/00275514.1982.12021470

[B26] Wells L. D. McManus P. S. (2013). A photographic diagnostic guide for identification of the principal cranberry fruit rot pathogens. Plant Health Prog. 14, 1. doi: 10.1094/PHP-2013-0729-01-DG

[B27] Wells L. D. Perry R. S. McManus P. S. (2014). Fungicide efficacy and specificity toward fungi in the cranberry fruit rot disease complex. Plant Health Prog. 15, 31–35. doi: 10.1094/PHP-RS-13-0024

[B28] Wells-Hansen L. D. McManus P. S. (2017). Year-to-year incidence of cranberry fruit rot and persistence of fungal pathogens. Plant Health Prog. 18, 114–119. doi: 10.1094/PHP-12-16-0073-RS

[B29] White T. J. Bruns T. Lee S. Taylor J. (1990). PCR Protocols: A Guide to Methods and Applications (San Diego, CA: Academic Press).

[B30] Wood B. McBride E. Nabetani K. Griffin T. Sabaratnam S. (2023). Prevalence and spatial distribution of cranberry fruit rot pathogens in British Columbia, Canada, and potential fungicides for fruit rot management. Front. Plant Sci. 14. doi: 10.3389/fpls.2023.1274094 PMC10667600 38023868

